# Intuitionistic Linguistic Multiple Attribute Decision-Making with Induced Aggregation Operator and Its Application to Low Carbon Supplier Selection

**DOI:** 10.3390/ijerph14121451

**Published:** 2017-11-24

**Authors:** Jun Liu, Xianbin Wu, Shouzhen Zeng, Tiejun Pan

**Affiliations:** 1 School of Management Science and Engineering, Nanjing University of Finance and Economics, Nanjing 210023, China; 9120031038@nufe.edu.cn; 2 Junior College, Zhejiang Wanli University, Ningbo 315100, China; 3 School of Business, Ningbo University, Ningbo 315211, China; 4 School of Information, Ningbo Dahongying University, Ningbo 315211, China; pantiejunmail@126.com

**Keywords:** intuitionistic linguistic set, induced aggregation operator, low carbon supplier selection, multiple attribute decision-making

## Abstract

The main focus of this paper is to investigate the multiple attribute decision making (MADM) method under intuitionistic linguistic (IL) environment, based on induced aggregation operators and analyze possibilities for its application in low carbon supplier selection. More specifically, a new aggregation operator, called intuitionistic linguistic weighted induced ordered weighted averaging (ILWIOWA), is introduced to facilitate the IL information. Some of its desired properties are explored. A further generalization of the ILWIOWA, called intuitionistic linguistic generalized weighted induced ordered weighted averaging (ILGWIOWA), operator is developed. Furthermore, by employing the proposed operators, a MADM approach based on intuitionistic linguistic information is presented. Finally, an illustrative example concerning low carbon supplier selection and comparative analyses are conducted to demonstrate the effectiveness and practicality of the proposed approach.

## 1. Introduction

Due to the increased consciousness on the issue of global warming and environmental protection, low carbon supply chain has been a popular research topic in recent years [1,2,3]. Identifying the suitable low carbon supplier is one of the most critical activities to achieve an efficient low-carbon supply chain, which plays a vital role in carbon emissions reduction and environmental sustainability [4,5]. In general, the supplier selection process is considered as a multiple attribute decision making (MADM) problem, since numerous and discordant attributes should be taken into account and evaluated [6,7,8,9]. In the literature, a variety of MADM approaches for supplier evaluation and selection have been proposed of which the prerequisites for using these methods is to assume that the information of attribute is clearly known and can be evaluated with exact values [10,11,12,13]. However, in real world problems, the rapid development of economics and business environment has made the decision progress of supplier selection more complex and uncertain. The intuitionistic fuzzy set (IFS), as proposed by Atanassov [14], is a very useful tool to describe and deal with such uncertainty and vagueness. A prominent characteristic of IFS is that it assigns to each element a membership degree and a non-membership degree. Due to its outstanding superiority, IFS has received more and more attention and has been widely applied in supplier evaluation and selection problems. For example, Sirbiladze and Badagadze [15] presented some IFS probabilistic aggregation operators to study the method of supplier selection. Büyüközkan and Göçer [16] developed an IFS MADM method based on axiomatic design methodology for the supplier selection problem. Yu [17] investigated the application of intuitionistic fuzzy Bonferroni mean approach in supplier selection problems. Krishankumar et al. [18] conducted a review on IF decision making process concerning supplier selection problems from 2013 to 2016, based on which, they presented a new MADM method to solve supplier selection problems. Tong and Wand [19] put forward the induced intuitionistic ordered weighted averaging (IIOWA) operator to solve a low carbon supplier selection with intuitionistic preference relations. More recently, Mehdi et al. [20] conducted a deep bibliometric survey on MADM methods for the evaluation and selection of suppliers from 2001 to 2016, which not only concentrates on an IF situation, but also focuses on other aspects of a fuzzy environment. 

From the existing literature, it can be seen that IFS is an effective tool to handle the problem of uncertainty and has been widely used in MADM including supplier evaluation and selection, investment selection, and other selection problems. However, sometimes it is difficult for decision makers to provide exact numbers for the membership and non-membership degrees of an IFS, while it is easy to provide linguistic assessment values in in real decision making. On the basis of the IFS and the linguistic assessment set, Wang and Li [21] proposed the concept of intuitionistic linguistic set (ILS), whose basic elements are intuitionistic linguistic numbers (ILNs). Since its appearance, the MADM method with ILS, especially the investigations of the intuitionistic linguistic (IL) aggregation method, have obtained more and more attention. For example, Wang and Li [21] proposed the expected values, score function, accuracy function, and some operational laws of the ILS. Based on the ordered weighted averaging (OWA) operator [22], Liu [23] developed some IL aggregation operators including the IL generalized dependent ordered weighted average (ILGDOWA) operator and the IL generalized dependent hybrid weighted aggregation (ILGDHWA) operator, respectively. Liu and Wang [24] proposed some IL power aggregation operators. Su et al. [25] extended the OWA distance (OWAD) operator to IL environment and presented the IL OWA distance (ILOWAD) operator. Xiao and Zhang [26] developed the IL inducing OWA (ILIOWA) operator, and studied its application in financial decision making problems.

How to deal with the evaluation information by using a proper aggregation operator is a critical step of the decision making. The induced OWA (IOWA) operator, as introduced by Yager and Filev [27], has been widely used to aggregate experts’ information in decision making, in which the ordering of arguments is induced by the order-inducing variables. The IOWA aggregates actual arguments coupled with the order-inducing variables. Depending upon its merit, a number of applications and extension of IOWA operator has been studied in the literature [28,29,30,31,32,33,34]. However, the IOWA operator cannot reflect the different magnitudes of the order-inducing variables, which often leads to a loss of information in the aggregation result. To circumvent this drawback, Manish [35] proposed an improved IOWA, called the weighted IOWA (weighted induced ordered weighted averaging (WIOWA)) operator, whose main advantage is its ability to take into account the inherent variations of the order-inducing variables. In the WIOWA operator, any characteristics that are associated with each attribute pair, such as preference, importance, and consistency can be treated as inducing variables. Moreover, he further extended the WIOWA operator to the intuitionistic fuzzy domain and studied its application in supplier selection problems.

Motivated by the idea of WIOWA operator, in this paper, we first propose the intuitionistic linguistic weighted induced ordered weighted averaging (ILWIOWA) operator, which is the extension of WIOWA by using the IL variables. Some desirable properties of the ILWIOWA operator are explored. We further develop the intuitionistic linguistic generalized weighted induced ordered weighted averaging (ILGWIOWA) operator, which provides a wide range of intuitionistic linguistic aggregation operators. At the same time, some special cases of the generalized parameters in these operators are analyzed. Finally, a method based on the proposed operator is developed and its application in low carbon supplier evaluation and selection is studied.

To do this, the remainder of this paper is organized as follows. In Section 2, we briefly review some basic concepts. Section 3 presents the ILWIOWA and ILGWIOWA operators and analyzes a wide range of particular cases. In Section 4, we develop a method for MADM based on the proposed operator and present an illustrative example in Section 5. Section 6 summarizes the main conclusions that are found in the paper.

## 2. Preliminaries

This section briefly reviews the intuitionistic linguistic set, the IOWA operator, and the WIOWA operator.

### 2.1. The Linguistic Set

For computational convenience, let $S=\left\{s_{\alpha }|\alpha =0,1,\ldots ,l-1\right\}$ be a finite and totally ordered discrete term set, where l is the odd value and $s_{\alpha }$ represents a possible value for a linguistic variable. For example, when l=7, a set S could be given as follows:

$$S=\{s_{0},s_{1},s_{2},s_{3},s_{4},s_{5},s_{6}\}=\{very\;poor,poor,slightly\;poor,fair,slightly\;good,good,very\;good\}$$

In these cases, it is usually required that there exist the following [36,37]:

(1) A negation operator: $\mathrm{Neg}(s_{i})=s_{l-i}$;
(2) The set is ordered: $s_{i}\leq s_{j}$ if and only if i≤j;
(3) Maximum operator: $\max(s_{i},s_{j})=s_{i}$, if i≥j;
(4) Minimum operator: $\min(s_{i},s_{j})=s_{i}$, if i≤j.

In order to preserve all the given information, Xu [37] extended the discrete term set S to a continuous term set $\bar{S}=\left\{s_{\alpha }|\alpha \in [0,l]\right\}$, where, if $s_{\alpha }\in S$, then we call $s_{\alpha }$ the original term, otherwise, we call $s_{\alpha }$ the virtual term. Consider any two linguistic terms $s_{\alpha },s_{\beta }\in \bar{S}$, and μ>0, the operations are defined as follows:

(1) $s_{\alpha }⊕s_{\beta }=s_{\alpha +\beta }$;
(2) $\mu s_{\alpha }=s_{\mu \alpha }$;
(3) $s_{\alpha }/s_{\beta }=s_{\alpha /\beta }$.

### 2.2. The Intuitionistic Linguistic Set (ILS)

The IFS theory is one of the most efficient tools to handle the vagueness and impreciseness in the data, which has received more and more attention from all over the world since its appearance [38,39,40,41,42,43]. In combining the advantage of the IFS and the linguistic terms, Wang and Li [21] first proposed the ILS, and gave the definition of ILS.

**Definition** **1** [21]**.** *An ILS A in* X *is defined as*

(1) $$A=\left\{\langle x\left[h_{\theta (x)},\left(\mu_{A}(x),v_{A}(x)\right)\right]\rangle |x\in X\right\}$$

here $h_{\theta (x)}\in \bar{S}$, and $\mu_{A}(x)$ and $v_{A}(x)$ represent the membership degree and non-membership degree of the element x related to linguistic index $h_{\theta (x)}$, respectively. $0\leq \mu_{A}(x)+v_{A}(x)\leq 1$, for all x∈X. For each ILS A in X, if

(2) $$\pi_{A}(x)=1-\mu_{A}(x)-v_{A}(x),\forall x\in X$$

then $\pi_{A}(x)$ is called the indeterminacy degree or hesitation degree of x to linguistic index $h_{\theta (x)}$.

For computational convenience, Wang and Li [21] further developed the definition of the intuitionistic linguistic number (ILN). An ILN is defined by $\tilde{a}=\langle s_{\theta (a)},\left(\mu (a),v(a)\right)\rangle$, where μ(a) and v(a), respectively, represent the membership and non-membership degrees of the linguistic variable $s_{\theta (x)}$ with μ(a),v(a)≥0, μ(a)+v(a)≤1. 

Let ${\tilde{a}}_{1}=\langle s_{\theta (a_{1})},\left(\mu (a_{1}),v(a_{1})\right)\rangle$ and ${\tilde{a}}_{2}=\langle s_{\theta (a_{2})},\left(\mu (a_{2}),v(a_{2})\right)\rangle$ be two ILNs and λ≥0, then the operations of ILNs are defined by [21]:

(1) ${\tilde{a}}_{1}+{\tilde{a}}_{2}=\langle s_{\theta (a_{1})+\theta (a_{2})},\left(1-\left(1-\mu (a_{1})\right)\left(1-\mu (a_{2})\right),v(a_{1})v(a_{2})\right)\rangle$;
(2) ${\tilde{a}}_{1}⊗{\tilde{a}}_{2}=\langle s_{\theta (a_{1})\times \theta (a_{2})},\left(\mu (a_{1})\mu (a_{2}),v(a_{1})+v(a_{2})-v(a_{1})v(a_{2})\right)\rangle$;
(3) $\lambda {\tilde{a}}_{1}=\langle s_{\lambda \theta (a_{1})},\left(1-{\left(1-\mu (a_{1})\right)}^{\lambda },{\left(v(a_{1})\right)}^{\lambda }\right)\rangle$.

For any ILN $\tilde{a}=\langle s_{\theta (a)},\left(\mu (a),v(a)\right)\rangle$, in [23] Liu defined the expectation function $E\left(\tilde{a}\right)$ by $s_{\theta (a)\times [\mu (a)+\frac{1}{2}\left(1-\mu (a)-v(a)\right)]}$ and introduced the accuracy function $H\left(\tilde{a}\right)=\frac{\theta (a)}{l-1}\times \left(\mu (a)+v(a)\right)$ to evaluate the accuracy degree of $\tilde{a}$. Further, he gave the following order relationship to compare ILNs.

**Definition** **2** [23]**.** *If* ${\tilde{a}}_{1}=\langle s_{\theta (a_{1})},\left(\mu (a_{1}),v(a_{1})\right)\rangle$ *and* ${\tilde{a}}_{2}=\langle s_{\theta (a_{2})},\left(\mu (a_{2}),v(a_{2})\right)\rangle$ *are any two ILNs, then:*

(1) *If* $E\left({\tilde{a}}_{1}\right)>E\left({\tilde{a}}_{2}\right)$ *, then* ${\tilde{a}}_{1}$ *is bigger than* ${\tilde{a}}_{2}$*, denoted by* ${\tilde{a}}_{1}>{\tilde{a}}_{2}$*;*
(2) *If* $E\left({\tilde{a}}_{1}\right)=E\left({\tilde{a}}_{2}\right)$*, and If* $H\left({\tilde{a}}_{1}\right)>H\left({\tilde{a}}_{2}\right)$*, then* ${\tilde{a}}_{1}>{\tilde{a}}_{2}$*, or, If* $H\left({\tilde{a}}_{1}\right)=H\left({\tilde{a}}_{2}\right)$*, then* ${\tilde{a}}_{1}={\tilde{a}}_{2}$*.*

### 2.3. The IOWA and WIOWA Operators

The main characteristic of the IOWA operator is that the reordering step is carried out with the order-inducing variables, but not with the values of the argument, which reflect a more complex reordering process [27]. It can be defined as follows:

**Definition** **3.** *An IOWA operator of dimension n is a mapping IOWA:* $R^{n}\times R^{n}\to R$ *that has an associated weighting* W *with* $w_{j}\in [0,1]$ *and* $\sum_{j=1}^{n}w_{j}=1$ *such that:*

(3) $$IOWA\left(\langle u_{1},a_{1}\rangle ,\ldots ,\langle u_{n},a_{n}\rangle \right)=\sum_{j=1}^{n}w_{j}b_{j}$$

where $b_{j}$ is that choice of the $a_{i}$ value for which the IOWA pair $\langle u_{i},a_{i}\rangle$ has the *j*th largest $u_{i}.$ Here $u_{i}$ is the order inducing variable and $a_{i}$ is the argument variable.

The WIOWA operator is a new extension of the IOWA operator, in which the order-inducing variables modify the associated weights. It can be defined as follows.

**Definition** **4** [35]**.** *A WIOWA operator of dimension n is a mapping WIOWA:* $R^{n}\times R^{n}\to R$ *that has an associated weighting* $W=(w_{1},w_{2},\ldots ,w_{n})$ *with* $w_{j}\in [0,1]$ *and* $\sum_{j=1}^{n}w_{j}=1$ *such that:*

(4) $$WIOWA\left(\langle u_{1},a_{1}\rangle ,\ldots ,\langle u_{n},a_{n}\rangle \right)=\sum_{j=1}^{n}v_{j}b_{j}$$

where $b_{j}$ is that choice of the $a_{i}$ value for which the WIOWA pair $\langle u_{i},a_{i}\rangle$ has the *j*th largest $u_{i}$. Here $u_{i}$ is the order inducing variable and $a_{i}$ is the argument variable. The weight $v_{j}$ is associated with $w_{j}\in W$ (j=1,2,…,n), as

(5) $$v_{j}=\frac{w_{j}u_{\sigma (j)}}{\sum_{j=1}^{n}w_{j}u_{\sigma (j)}}$$

where (σ(1),σ(2),…,σ(n)) is any permutation of (1,2,…,n) for which $u_{\sigma (j-1)}\geq u_{\sigma (j)}$ for all j>1.

The WIOWA is suitable to deal with exact numbers rather than other types of arguments. Manish [35] extended it to intuitionistic fuzzy environment. However, there is no study on applications of the WIOWA with an ILS, although the use of ILS is proven to be a more powerful tool to handle uncertain information than the use of IFS. Therefore, in what follows, we shall extend the WIOWA operator to an IL environment and study its application in MADM problems that are related to supplier selection.

## 3. Intuitionistic Linguistic WIOWA Operator

The ILWIOWA operator is an extension of the WIOWA operator by using uncertain information represented in the form of ILNs. Therefore, it uses the main characteristics of the WIOWA operator that are associated weights that are closely related to the order-inducing variables. Thus, this operator enables us to capture the variations in the order-inducing variables in the final result. It can be defined as follows.

**Definition** **5.** *Let* ${\tilde{a}}_{i}=\langle s_{\theta_{i}},\left(\mu_{i},v_{i}\right)\rangle$ (i=1,2,…,n) *be a collection of ILNs, an ILWIOWA operator of dimension* n *is a mapping ILWIOWA:* $\Omega^{n}\times \Omega^{n}\to \Omega$ *defined by* $W=(w_{1},w_{2},\ldots ,w_{n})$ *and* $u=(u_{1},u_{2},\ldots ,u_{n})$ *such that:*

(6) $$ILWIOWA\left(\langle u_{1},{\tilde{a}}_{1}\rangle ,\ldots ,\langle u_{n},{\tilde{a}}_{n}\rangle \right)=\sum_{j=1}^{n}v_{j}{\tilde{b}}_{j}$$

where ${\tilde{b}}_{j}$ is ${\tilde{a}}_{i}$ value of the ILWIOWA pair $\langle u_{i},{\tilde{a}}_{i}\rangle$ having the jth largest $u_{i}$, and the weight $v_{j}$ associated with $w_{j}\in W$ (j=1,2,…,n), is defined as Equation (5).

Next, we give a simple numerical example showing how to use the ILWIOWA operator in an aggregation process.

**Example** **1.** *Let the argument to be aggregated in the form* $\left(u_{i},{\tilde{a}}_{i}\right)$ *be* $(\left(8,\langle s_{6},(0.5,0.4)\rangle \right),\left(7,\langle s_{4},(0.3,0.4)\rangle \right),\left(9,\langle s_{4},(0.6,0.3)\rangle \right),\left(5,\langle s_{3},(0.2,0.6)\rangle \right))$*, and the ILN* ${\tilde{a}}_{i}$ *is defined in a seven linguistic terms set* $S=\{s_{0},s_{1},s_{2},s_{3},s_{4},s_{5},s_{6}\}$*. First we should take a record of the argument according to the values* $u_{i}(i=1,2,3,4)$ *to get:*

$$(\left(9,\langle s_{4},(0.6,0.3)\rangle \right),\left(8,\langle s_{6},(0.5,0.4)\rangle \right),\left(7,\langle s_{4},(0.3,0.4)\rangle \right),\left(5,\langle s_{3},(0.2,0.6)\rangle \right))$$

Assume the following weighting vector W=(0.4,0.3,0.2,0.1), then, we calculate the weight $v_{j}$(j=1,2,3,4):

$$v_{1}=\frac{0.4\times 9}{\left(0.4\times 9)+(0.3\times 8)+(0.2\times 7)+(0.1\times 5\right)}=0.456$$

Similarly, we can get

$$v_{2}=0.304,v_{3}=0.177,v_{4}=0.063$$

We apply the ILWIOWA operator in (6) to perform the aggregation as follows:

$$ILWIOWA(\left(8,\langle s_{6},(0.5,0.4)\rangle \right),\left(7,\langle s_{4},(0.3,0.4)\rangle \right),\left(9,\langle s_{4},(0.6,0.3)\rangle \right),\left(5,\langle s_{3},(0.2,0.6)\rangle \right))=0.456⊗\langle s_{4},(0.6,0.3)\rangle ⊕0.304⊗\langle s_{6},(0.5,0.4)\rangle ⊕0.177⊗\langle s_{4},(0.3,0.4)\rangle ⊕0.063⊗\langle s_{3},(0.2,0.6)\rangle =\langle s_{4.545},(0.506,0.360)\rangle$$

In order to make a comparison of the (previous) ILWIOWA aggregation result with that of the corresponding intuitionistic linguistic IOWA (ILIOWA) operator [26], the corresponding aggregation result is required. This aggregation result reads to follows:

$$ILIOWA(\left(8,\langle s_{6},(0.5,0.4)\rangle \right),\left(7,\langle s_{4},(0.3,0.4)\rangle \right),\left(9,\langle s_{4},(0.6,0.3)\rangle \right),\left(5,\langle s_{3},(0.2,0.6)\rangle \right))=0.4⊗\langle s_{4},(0.6,0.3)\rangle ⊕0.3⊗\langle s_{6},(0.5,0.4)\rangle ⊕0.2⊗\langle s_{4},(0.3,0.4)\rangle ⊕0.1⊗\langle s_{3},(0.2,0.6)\rangle =\langle s_{4.5},(0.487,0.371)\rangle$$

As we can see, when compared with the ILIOWA operator, besides inducing the order among the arguments, the order-inducing variable values in the ILWIOWA operator also play an important role in associated weights. 

Based on the operational laws of the ILNs described earlier, we can derive the result, as shown as Theorem 1.

**Theorem** **1.** *Let* ${\tilde{a}}_{i}=\langle s_{\theta_{i}},\left(\mu_{i},v_{i}\right)\rangle$ (i=1,2,…,n) *be a collection of ILNs, the resulting aggregated value by the ILWIOWA is still an ILN.*

**Theorem** **2.** *(Idempotency). If all* ${\tilde{a}}_{i}=\langle s_{\theta_{i}},\left(\mu_{i},v_{i}\right)\rangle$ (i=1,2,…,n) *are equal, i.e.,* ${\tilde{a}}_{i}=a=\langle s_{\theta },\left(\mu ,v\right)\rangle$ *for all* i*, then*

(7) $$ILWIOWA\left(\langle u_{1},{\tilde{a}}_{1}\rangle ,\ldots ,\langle u_{n},{\tilde{a}}_{n}\rangle \right)=\tilde{a}$$

**Theorem** **3.** *(Monotonicity). Let* $\left({\tilde{a}}_{1},{\tilde{a}}_{2},\ldots ,{\tilde{a}}_{n}\right)$ *and* $\left({\tilde{a}}_{1}^{\prime },{\tilde{a}}_{2}^{\prime },\ldots ,{\tilde{a}}_{n}^{\prime }\right)$ (i=1,2,…,n) *be two collection of ILNs, if* ${\tilde{a}}_{i}^{\prime }\leq {\tilde{a}}_{i}$ *for all* i*, then*

(8) $$ILWIOWA\left(\langle u_{1},{\tilde{a}}_{1}\rangle ,\ldots ,\langle u_{n},{\tilde{a}}_{n}\rangle \right)\leq ILWIOWA\left(\langle u_{1},{\tilde{a}}_{1}^{\prime }\rangle ,\ldots ,\langle u_{n},{\tilde{a}}_{n}^{\prime }\rangle \right)$$

**Theorem** **4.** *(Boundedness). The ILWIOWA operator lies between the max and min operators, i.e.,*

(9) $$\min({\tilde{a}}_{1},{\tilde{a}}_{2},\ldots ,{\tilde{a}}_{n})\leq ILWIOWA\left(\langle u_{1},{\tilde{a}}_{1}\rangle ,\ldots ,\langle u_{n},{\tilde{a}}_{n}\rangle \right)\leq \max({\tilde{a}}_{1},{\tilde{a}}_{2},\ldots ,{\tilde{a}}_{n})$$

**Theorem** **5.** *(Permutation) Let* $\left(\langle u_{1},{\dot{\tilde{a}}}_{1}\rangle ,\ldots ,\langle u_{n},{\dot{\tilde{a}}}_{n}\rangle \right)$ (i=1,2,…,n) *is any permutation of* $\left(\langle u_{1},{\tilde{a}}_{1}\rangle ,\ldots ,\langle u_{n},{\tilde{a}}_{n}\rangle \right)$

(10) $$ILWIOWA\left(\langle u_{1},{\tilde{a}}_{1}\rangle ,\ldots ,\langle u_{n},{\tilde{a}}_{n}\rangle \right)=ILWIOWA\left(\langle u_{1},{\dot{\tilde{a}}}_{1}\rangle ,\ldots ,\langle u_{n},{\dot{\tilde{a}}}_{n}\rangle \right)$$

Note that the proofs of these theorems are straightforward and thus omitted. Moreover, some particular cases of ILWIOWA operator can be explored following the vein of recent literature [28,40,44]. Especially, if $u_{1}=u_{2}=\ldots =u_{n}=1$, then the ILWIOWA operator is reduced to the ILOWA operator [26].

Next, we shall develop an extension of the ILWIOWA operator by using the generalized means approach [28], therefore we get a new IL aggregation operator, called the IL generalized WIOWA (ILGWIOWA) operator. The main advantage of this operator is that it includes a wide range of IL aggregation operators. Thus, we have more chance to select the alternative that best fits with our interests. The ILGWIOWA operator can be defined as follows.

**Definition** **6.** *Let* ${\tilde{a}}_{i}=\langle s_{\theta_{i}},\left(\mu_{i},v_{i}\right)\rangle$ (i=1,2,…,n) *be a collection of ILNs, an ILGWIOWA operator of dimension* n *is a mapping ILGWIOWA:* $\Omega^{n}\times \Omega^{n}\to \Omega$ *defined by* $W=(w_{1},w_{2},\ldots ,w_{n})$ *and* $u=(u_{1},u_{2},\ldots ,u_{n})$ *such that:*

(11) $$ILGWIOWA\left(\langle u_{1},{\tilde{a}}_{1}\rangle ,\ldots ,\langle u_{n},{\tilde{a}}_{n}\rangle \right)={\left(\sum_{j=1}^{n}v_{j}{\left({\tilde{b}}_{j}\right)}^{\lambda }\right)}^{1/\lambda }$$

where ${\tilde{b}}_{j}$ is ${\tilde{a}}_{i}$ value of the ILGWIOWA pair $\langle u_{i},{\tilde{a}}_{i}\rangle$ having the *j*th largest $u_{i}$, and the weight $v_{j}$ associated with $w_{j}\in W$ (j=1,2,…,n), is defined as Equation (5). λ is a parameter such that λ∈(−∞,+∞)−{0}.

It is easy to prove that the ILGWIOWA operator has properties of idempotency, boundedness, monotonicity, and commutativity. Moreover, this model covers a wide range of IL aggregation operators, for example,

- If λ→0, we form the IL weighted induced ordered weighted geometric (ILWIOWG) operator.
- If *λ* = 1, we get the ILWIOWA operator, and the same time we obtain a kind of particular cases of the ILWIOWA.
- If *λ* = 2, we get the IL weighted induced ordered weighted quadratic averaging (ILWIOWQA) operator.
- If λ=−1, the IL weighted induced ordered weighted harmonic averaging (ILWIOWHA) operator is obtain.
- Etc.

## 4. Multiple Attribute Decision Making Based on the ILGWIOWA Operator 

As a general framework of induced aggregation operators, the ILGWIOWA operator can be applied in many areas, such as decision theory, statistics, and economics. In this section, we study the application of ILGWIOWA operator in MADM problems concerning the supplier selection in supply chain management. For a MADM problem, let $A=\left\{A_{1},A_{2},\ldots ,A_{m}\right\}$ be a discrete set of alternatives, and let $G=\left\{G_{1},G_{2},\ldots ,G_{n}\right\}$ be the set of attributes. The main steps of selection best decision variant(s) are as follows.

Step 1. Form the IL evaluation matrix $A={\left({\tilde{\alpha }}_{{}_{ij}}^{}\right)}_{m\times n}$, where ${\tilde{\alpha }}_{{}_{ij}}^{}$ is a preference value provided by the experts for alternative $A_{i}$ with respect to the attribute $C_{j}$, which takes the form of an ILN.

Step 2. Calculate the order-inducing variable values $u=(u_{1},u_{2},\ldots ,u_{n})$, which could be any important property values concerning the alternatives, such as belief levels, consistency, and importance. In this paper, we assume that the order-inducing variable values represent the experts’ belief levels of the evaluations.

Step 3. Utilize the ILGWIOWA operator to aggregate the evaluations ${\tilde{\alpha }}_{{}_{ij}}^{}$ for each alternative $A_{i}$.

(12) $${\tilde{\alpha }}_{i}=ILGWIOWA\left(<u_{1},{\tilde{\alpha }}_{i1}>,\ldots ,<u_{n},{\tilde{\alpha }}_{in}>\right),i=1,2,\ldots ,m$$

Note that we can also use a wide range of particular cases of the ILGWIOWA operators to aggregate the evaluations in this step according to our interesting.

Step 4. Rank all of the alternatives $A_{i}$ (i=1,2,…,m) and identify the optimal one(s) in accordance with ${\tilde{\alpha }}_{i}$ (i=1,2,…,m).

## 5. An Example of Low Carbon Supplier Selection

In recent years, the increasing of carbon emissions has threatened public health and economic development mode. This challenge has prompted governments and companies worldwide to perform attempt in order to increase and stimulate investments in low carbon economics. In the decision process, a crucial stage is to select the suitable low carbon suppliers. What is more, this process involves multiple requirements that are associated with uncertain information, all of which have to be taken into account and assessed simultaneously. Therefore, the selection of low carbon supplier is a highly vague and complex decision process. Tong and Wang [19] and Lin and Wang [45] investigated the selection of low carbon supplier using IF and linguistic MADM method, respectively. However, the linguistic set and IFS can be viewed as special cases of ILS. Therefore, ILS can be used to describe the linguistic set and IF information in this category of low carbon supplier selection problems, reflecting the real-world situations more accurately. To address this important issue, in this section, we present a numerical example on the use of the developed operator in a MADM problem aimed at the low carbon supplier selection.

To select an appropriate low carbon supplier for a manufacturer, the committee of decision makers offer their assessments to four potential suppliers $A_{i}$(i=1,2,…,4) based on a set of criteria accounting for low carbon technology ($C_{1}$), cost ($C_{2}$), risk factor ($C_{3}$), and capacity ($C_{4}$) (adapted from [19]). It is assumed that the four possible alternatives are evaluated using the linguistic term set $S=(s_{1},s_{2},s_{3},s_{4},s_{5},s_{6},s_{7})$ by decision makers under the above four attributes, and construct, respectively, the intuitionistic linguistic evaluation matrix $A^{}={\left({\tilde{a}}_{{}_{ij}}^{}\right)}_{4\times 4}$ as shown in Table 1.

After evaluating, the decision makers present their order-inducing variable values $u=(u_{1}=9,u_{2}=8,u_{3}=6,u_{4}=7)$, which represent their confidence levels of the evaluation. Suppose that the weighting vector W=(0.4,0.3,0.2,0.1). By exploiting this information, we can utilize the ILGWIOWA operator (suppose λ=1) to derive the collective overall preference values ${\tilde{a}}_{i}{}_{}$ that are associated with each of the alternative $A_{i}$(i=1,2,3,4):

$${\tilde{a}}_{1}=\langle s_{3.75},0.501,0.404\rangle ,\;{\tilde{a}}_{2}=\langle s_{4.225},0.465,0.481\rangle ,\;{\tilde{a}}_{3}=\langle s_{3.725},0.422,0.548\rangle ,\;{\tilde{a}}_{4}=\langle s_{3.975},0.494,0.410\rangle$$

Calculate the expected values $E({\tilde{a}}_{i})$(i=1,2,3,4) of the collective overall IL preference values ${\tilde{a}}_{i}$(i=1,2,3,4),

$$E({\tilde{a}}_{1})=s_{2.056},\;E({\tilde{a}}_{2})=s_{2.078},\;E({\tilde{a}}_{3})=s_{1.629},\;E({\tilde{a}}_{4})=s_{2.128}$$

Rank all of the alternatives $A_{i}$(i=1,2,3,4) in accordance with the expected values $E({\tilde{a}}_{i})$ of the collective overall IL preference values ${\tilde{a}}_{i}$(i=1,2,3,4), we can get:

$$A_{4}≻A_{2}≻A_{1}≻A_{3},$$

and thus the most desirable alternative is $A_{4}$.

It is interesting to study the validation and comparison of results with other IL aggregation operators. In this example, we compare the results of the ILGWIOWA with the ILIOWA operator, in which the order-inducing variables is only used to the order inducing step, but is not explicitly used in the aggregation. The aggregation results of ILIOWA operator are:

$${\tilde{a}}_{1}=\langle s_{3.7},0.496,0.407\rangle ,\;{\tilde{a}}_{2}=\langle s_{4.2},0.482,0.480\rangle ,$$

$${\tilde{a}}_{3}=\langle s_{3.8},0.423,0.540\rangle ,\;{\tilde{a}}_{4}=\langle s_{3.8},0.512,0.411\rangle .$$

Therefore, the expected values $E({\tilde{a}}_{i})$ of the collective overall values ${\tilde{a}}_{i}$(i=1,2,3,4),

$$E({\tilde{a}}_{1})=s_{2.014},\;E({\tilde{a}}_{2})=s_{2.150},\;E({\tilde{a}}_{3})=s_{1.677},\;E({\tilde{a}}_{4})=s_{2.091}$$

The ordering of the alternatives according to the decreasing order of $E({\tilde{a}}_{i})$

$$A_{2}≻A_{4}≻A_{1}≻A_{3},$$

and thus the most desirable alternative is $A_{2}$.

As we can, see we get a different ranking order of the alternatives by using the ILIOWA operator. The reason being that the role of the order-inducing variables in the ILIOWA operator is limited to the order-inducing, which leads to a loss of the intrinsic variation information and a biased aggregation result. 

Furthermore, it is possible to explore how the parameter λ of the ILGWIOWA operator affects the aggregation results. We obtain some integrated results and the ranking of the alternatives by using some key particular cases of the ILGWIOWA operators, as shown in Table 2.

As we can see, depending on the used parameter value λ, the ordering of the alternatives may be different. The rule of the parameter λ in the ILGWIOWA operator is to describe certain aspects of attitude of the decision makers, and as such the aggregation results are more reasonable and correspond better to real-world situations. Note that this method is rather flexible as it allows the decision maker(s) for more choices of the aggregation schemes by assigning the different values to the parameters. Therefore, the decision maker will select for his decision the one that best fits with his or her interests or beliefs.

## 6. Conclusions

In this paper, we have proposed two new IL induced aggregation operators, called the IWIOWA and ILGWIOWA operator, respectively. The prominent characteristic of the developed operators is that the order-inducing variables do not only induce the order of arguments, but they also moderate the corresponding weight vectors. We have also introduced a method for tackling IL MADM problems by means of the proposed operators. It is shown that the developed operators are highly appealing in the sense that they enable us to aggregate the intuitionistic linguistic preferences of experts in the analysis, thus providing more robust conclusions. Finally, a numerical example of selecting the low carbon supplier is provided to illustrate the solution processes and demonstrate the feasibility of the proposed procedure. It is observed that the method is particularly attractive as it allows for a higher degree of flexibility when specifying the parameter in accordance with the problem context and decision makers’ interests. 

In future work, we would like to extend this method in terms of the use of the distance measures and Pythagorean fuzzy set [46,47]. One can also consider the different decisional problematics based on the proposed tools and procedures, such as the choice, the ranking, or the sorting problematic [48].

## Figures and Tables

**Table 1:** Intuitionistic linguistic evaluation matrix.

	$C_{1}$	$C_{2}$	$C_{3}$	$C_{4}$
$A_{1}$	$\langle s_{5},(0.6,0.3)\rangle$	$\langle s_{2},(0.4,0.6)\rangle$	$\langle s_{5},(0.7,0.2)\rangle$	$\langle s_{3},(0.2,0.6)\rangle$
$A_{2}$	$\langle s_{4},(0.4,0.6)\rangle$	$\langle s_{5},(0.4,0.5)\rangle$	$\langle s_{3},(0.8,0.1)\rangle$	$\langle s_{4},(0.5,0.5)\rangle$
$A_{3}$	$\langle s_{3},(0.5,0.5)\rangle$	$\langle s_{4},(0.3,0.7)\rangle$	$\langle s_{4},(0.7,0.2)\rangle$	$\langle s_{5},(0.2,0.7)\rangle$
$A_{4}$	$\langle s_{6},(0.5,0.4)\rangle$	$\langle s_{2},(0.3,0.6)\rangle$	$\langle s_{3},(0.9,0.1)\rangle$	$\langle s_{3},(0.4,0.5)\rangle$

**Table 2:** Aggregated results by the intuitionistic linguistic generalized weighted induced ordered weighted averaging (ILGWIOWA) and the rankings of alternatives.

	$A_{1}$	$A_{2}$	$A_{3}$	$A_{4}$	Ranking
ILGIWIOWA (*λ* = 2)	$s_{2.003}$	$s_{2.215}$	$s_{1.887}$	$s_{2.224}$	$A_{4}≻A_{2}≻A_{1}≻A_{3}$
ILGIWIOWA (*λ* = 3)	$s_{1.926}$	$s_{2.245}$	$s_{1.725}$	$s_{2.312}$	$A_{4}≻A_{2}≻A_{1}≻A_{3}$
ILGIWIOWA (λ→0)	$s_{2.234}$	$s_{2.125}$	$s_{1.745}$	$s_{2.035}$	$A_{1}≻A_{2}≻A_{4}≻A_{3}$
ILGIWIOWA (*λ* = −1)	$s_{1.836}$	$s_{2.234}$	$s_{1.756}$	$s_{2.033}$	$A_{2}≻A_{4}≻A_{1}≻A_{3}$
